# Ambient and Microenvironmental Particles and Exhaled Nitric Oxide Before and After a Group Bus Trip

**DOI:** 10.1289/ehp.9386

**Published:** 2006-12-04

**Authors:** Sara Dubowsky Adar, Gary Adamkiewicz, Diane R. Gold, Joel Schwartz, Brent A. Coull, Helen Suh

**Affiliations:** 1 Department of Environmental and Occupational Health Sciences, University of Washington, Seattle, Washington, USA; 2 Department of Environmental Health, Harvard School of Public Health, Boston, Massachusetts, USA; 3 Channing Laboratory, Department of Medicine, Brigham and Women’s Hospital, Boston, Massachusetts, USA; 4 Department of Epidemiology; 5 Department of Biostatistics, Harvard School of Public Health, Boston, Massachusetts, USA

**Keywords:** air pollution, exhaled nitric oxide, inflammation, particulate matter, traffic

## Abstract

**Objectives:**

Airborne particles have been linked to pulmonary oxidative stress and inflammation. Because these effects may be particularly great for traffic-related particles, we examined associations between particle exposures and exhaled nitric oxide (FE_NO_) in a study of 44 senior citizens, which involved repeated trips aboard a diesel bus.

**Methods:**

Samples of FE_NO_ collected before and after the trips were regressed against microenvironmental and ambient particle concentrations using mixed models controlling for subject, day, trip, vitamins, collection device, mold, pollen, room air nitric oxide, apparent temperature, and time to analysis. Although ambient concentrations were collected at a fixed location, continuous group-level personal samples characterized microenvironmental exposures throughout facility and trip periods.

**Results:**

In pre-trip samples, both microenvironmental and ambient exposures to fine particles were positively associated with FE_NO_. For example, an interquartile increase of 4 μg/m^3^ in the daily microenvironmental PM_2.5_ concentration was associated with a 13% [95% confidence interval (CI), 2–24%) increase in FE_NO_. After the trips, however, FE_NO_ concentrations were associated pre-dominantly with microenvironmental exposures, with significant associations for concentrations measured throughout the whole day. Associations with exposures during the trip also were strong and statistically significant with a 24% (95% CI, 15–34%) increase in FE_NO_ predicted per interquartile increase of 9 μg/m^3^ in PM_2.5_. Although pre-trip findings were generally robust, our post-trip findings were sensitive to several influential days.

**Conclusions:**

Fine particle exposures resulted in increased levels of FE_NO_ in elderly adults, suggestive of increased airway inflammation. These associations were best assessed by microenvironmental exposure measurements during periods of high personal particle exposures.

Numerous epidemiologic studies have linked particulate air pollution to increased morbidity and mortality. Observed increases in acute exacerbations of pulmonary and cardiovascular disease are thought to be partly the result of an inflammatory cascade that is triggered by oxidative stress to the lungs (Seaton et al. 1995). Existing research supports this hypothesized mechanism with associations documented between particulate exposures and increases in oxidative radicals in the lung (Gurgueira et al. 2002), enhanced production of proinflammatory cytokines by alveolar macrophages (van Eeden et al. 2001), and influxes of inflammatory cells into the lungs (Ghio et al. 2000). Particulate levels have also been linked to clinical effects such as respiratory-related symptoms (Ward and Ayres 2004), medical visits (Schwartz et al. 1993), and death (Clancy et al. 2002; Samet et al. 2000).

Adverse health effects have been documented for pollution of nonspecific origin as well as pollution from mobile sources. Findings with traffic-related pollutants have been reasonably consistent despite the fact that most investigations used relatively crude measures of traffic, such as proximity to traffic (Garshick et al. 2003; Janssen et al. 2003; Nitta et al. 1993; van Vliet et al. 1997; Venn et al. 2005), traffic density (Behrens et al. 2004; Ciccone et al. 1998; Janssen et al. 2003; Nicolai et al. 2003; van Vliet et al. 1997; Wjst et al. 1993), and estimated and measured levels of ambient traffic-related pollutants (Brauer et al. 2002; Hwang et al. 2005; Janssen et al. 2003; Kim et al. 2004; Migliaretti et al. 2005; van Vliet et al. 1997). Most of these investigations have been limited to children, however, with few investigations specifically examining the effects of traffic-related pollutants in the elderly, a population believed to have enhanced susceptibility with respect to air pollution (Bateson and Schwartz 2004; Goldberg et al. 2001; Katsouyanni et al. 2001).

For this article, we examined pulmonary inflammation in an elderly cohort as a result of direct exposures to traffic-related pollution. Specifically, we measured fractional concentrations of exhaled nitric oxide (FE_NO_) because this is generally considered to be an effective noninvasive marker for assessing subclinical pulmonary inflammation (American Thoracic Society 2006; van Amsterdam et al. 2000). Data for this investigation come from a study designed specifically to enhance the magnitude and variability of exposures to traffic. Our design involved transporting senior citizens from their suburban living facilities into urban areas through field trips aboard a diesel-powered bus. Using the resulting enhanced exposures, we investigated the associations between particulate air pollution and FE_NO_ in samples collected before and after the group trips. We hypothesized that increasing levels of particulate air pollution would be associated with increasing levels of FE_NO_ for all samples. We further hypothesized that stronger associations would be seen in samples collected after the trip than samples collected before the trip, because of either enhanced toxicity of traffic-related particles and/or decreased measurement error.

## Methods

### Study population

Data were collected from 44 nonsmoking seniors (≥ 60 years of age) under the supervision of the Harvard School of Public Health Human Subjects Committee. All participants were independently mobile and lived in one of four independent senior residences in suburban St. Louis, Missouri. Because data on heart rate variability also were collected in this study, individuals with atrial flutter, atrial fibrillation, or a paced rhythm were excluded from participation. Similarly, individuals with left bundle branch blocks were selected only if their heart rate variability could be ascertained. Persons with unstable angina also were excluded. All participants provided informed written consent.

### Study design

All study subjects were asked to participate in four group trips into downtown St. Louis between March and June 2002. Each subject was scheduled to participate approximately once per month in an outing that included one activity, lunch, and two standardized 1-hr periods aboard a diesel shuttle bus. Activities used in this study included a theater performance, Omni movie, outdoor band concert, and a Mississippi River boat cruise. All trips typically begin around 0930 and concluded by 1600 hr.

Samples of FE_NO_ were collected between 0800 and 0900 hr on the mornings before and after each trip. In the hours surrounding these samples, group-level measurements of particle concentrations also were collected using several continuous instruments installed on two portable carts. These carts were first positioned in a central location inside the participants’ living facilities 24 hr before each trip. The carts remained at the facilities until it was time for the trips, at which point they followed the participants from the health testing room, onto the bus, to the group activity, and to lunch. After the trip home aboard the bus, the carts were returned to the central location in the living facility where they remained until the conclusion of the health testing on the following morning. Continuous measurements of ambient particles and gases also were collected from a central monitoring station in East St. Louis, Illinois.

### Health measurements

Exhaled breath samples were collected on the mornings before and after each trip in accordance with the American Thoracic Society guidelines for offline measurements of FE_NO_ (American Thoracic Society 2006). To remove any nitric oxide in inspiratory air, all participants began FE_NO_ sampling by first tidal breathing through a nitric oxide scrubber for a minimum of 30 sec. Immediately following this breathing exercise, participants inspired to total lung capacity and exhaled without delay into a Mylar bag at a constant pressure of 12.5 mmHg. This oropharyngeal pressure was used to minimize sampling variability by preventing nasal nitric oxide interference and normalizing the expiratory flow rates to approximately 350 mL/sec (American Thoracic Society 2006). Participants also wore nose clips and were instructed to form a tight seal around the mouthpiece to prevent any inadvertent contamination by room air. Subjects were coached by trained technicians throughout each maneuver to ensure accurate FE_NO_ collection. To counter-act any subtle deviations from the collection protocol, three sequential samples were collected per person per session. Room air samples also were collected during each sampling period to assess the level of ambient nitric oxide levels at the time of breath collection. Similar samples were taken in the laboratory where FE_NO_ was analyzed within 24 hr of collection using a model 42 Chemiluminescence Analyzer (Thermo Electron Corporation, Franklin, MA). In our analyzis we used the median value of each set of three balloons as the measure of central tendency that was least sensitive to outlying values. Samples with insufficient volume to obtain a stable chemical measurement were excluded from this analysis.

Immediately preceding the collection of FE_NO_ samples, information regarding health status and medication use was ascertained using daily technician-administered questionnaires. In addition, data regarding the participants’ overall health status were collected at baseline before enrollment in the study.

### Exposure measurements

Two portable carts containing continuous air pollution monitors were used to measure group-level microenvironmental exposures to traffic-related pollutants, including fine particulate mass (< 2.5 μm aerodynamic diameter; PM_2.5_), black carbon, and size-specific particle counts. PM_2.5_ concentrations were measured continuously using a DustTrak aerosol monitor model 8520 (TSI, Shoreview, MN) with a Nafion diffusion dryer (Perma Pure LLC, Toms River, NJ). Integrated samples of PM_2.5_ mass also were collected using a Harvard Impactor (Air Diagnostics and Engineering Inc., Harrison, ME) for daily calibration of the trip and facility periods. Continuous black carbon concentrations were measured using a portable aethalometer (Magee Scientific, Berkeley, CA) with a 2.5-μm impaction inlet. Particle counts were measured using a model CI500 optical particle counter (Climet Instruments Company, Redlands, CA) with a modified flow rate of 0.1 cubic feet per minute. Before analysis, data from the Climet were aggregated by aerodynamic diameter and expressed as fine (0.3–2.5 μm) and coarse (2.5–10 μm) particle count concentrations. Temperature and relative humidity were recorded with a Telaire model 7001 carbon dioxide monitor (General Electric Sensing, Goleta, CA) and used to calculate apparent temperature, a biological weather stress index that incorporates both temperature and relative humidity (O’Neill et al. 2003).

Ambient particle data were obtained from a U.S. Environmental Protection Agency–(EPA)–sponsored Supersite. This Supersite is located in an industrial area adjacent to several interstate highways and just across the Mississippi River from downtown St. Louis. From its location approximately 10–15 miles from our facilities, concentrations of ambient PM_2.5_ were recorded using a continuous ambient mass monitor (Andersen Instruments, Smyrna, Georgia) with a Nafion diffusion dryer. Ambient black carbon concentrations were reported using an aethalometer (McGee Scientific). Criteria gas data were obtained from the Missouri Department of Natural Resources and Illinois EPA monitoring station, located immediately adjacent to the Supersite (http://www.epa.gov/ttn/airs/air-saqs/detaildata/downloadaqsdata.htm). These measurements were collected using a model 48 carbon monoxide analyzer (Thermo Electron Corporation), a model 2000A nitrogen dioxide analyzer (API, San Diego, California), a model 4108 sulfur dioxide analyzer (Dasibi Environmental Corporation, Glendale, California), and a model 1008RS ozone analyzer (Dasibi Environmental Corporation). Daily pollen and mold data also were obtained from the county health department and examined as total counts.

We calculated mean concentrations for all exposure metrics over the 6 and 24 hr preceding each FE_NO_ sample. These averaging periods were selected to maximize data completeness and were based on the findings of past investigations, which have indicated that the strongest associations between exhaled nitric oxide and particles occur within the first 24 hr (Adamkiewicz et al. 2004; Fischer et al. 2002; Steerenberg et al. 2001). In addition, associations between exposures measured during the trip periods were examined with post-trip FE_NO_ levels.

### Statistical analysis

Before statistical modeling, all measures of FE_NO_ were log-transformed because the data were highly skewed. We then ran mixed models containing random subject effects using SAS (version 8.02; SAS Institute, Cary, NC) to examine the impact of various personal characteristics on FE_NO_. We calculated empirical (sandwich) standard errors to ensure conclusions were robust to choice of covariance model. We modeled absolute differences between FE_NO_ levels on days before and after the trip using a mixed model with random intercepts; and we used an analysis of variance model with autoregressive terms to distinguish between the mean exposure levels on pre-trip and post-trip days. Spearman correlations also were calculated to investigate the relationships between the exposure metrics.

We examined associations between FE_NO_ and air pollution using mixed models with random subject effects in SAS. Time-varying parameters evaluated as potential confounders included the categorical variables of day of week, trip type, FE_NO_ collection device, current illness, as well as the use of vitamins, antihistamines, statins, steroids, and asthma medications. We also investigated continuous predictors of apparent temperature, pollen, mold, nitric oxide levels in the testing room, and the time between sample collection and analysis. Of these covariates, day of week, trip, collection device, vitamin use, pollen, mold, apparent temperature during the current hour, time to analysis, and nitric oxide concentrations in the testing room were selected for inclusion in our models. These covariates were selected based on their significant relationship with FE_NO_ in either pre-trip or post-trip samples at the 0.2 level among the unexposed (lower 50th percentile by exposure) (Mickey and Greenland 1989). All confounders were treated as categorical or linear except for apparent temperature, which was modeled as a linear spline with one knot. This more flexible form allowed for the nonlinear trend observed with apparent temperature using LOESS smoothing plots from S-Plus2000 (MathSoft, Cambridge, MA). This form was considered reasonable based on our belief that temperatures above or below some ideal level may produce unfavorable physiologic consequences. All pollutants were assumed to have a linear relationship with FE_NO_.

Our models first examined the single-pollutant associations with FE_NO_ samples collected before the trips and then with FE_NO_ samples collected after the trips. Pre- and post-trip samples were analyzed separately (i.e., change analyses were not performed) based on the anticipated reduction in statistical power due to increased missing data and numbers of confounders required in a single model (i.e., 2 days of confounders per change score). Change score models also would not allow us to examine differences in effect sizes between days with and without bus trips.

Our main models examined associations between FE_NO_ levels and the 6- and 24-hr averages of pollution concentrations preceding the health measurements. In each of these analyses, we compared the associations for microenvironmental and ambient particles to provide insight about the level of measurement error introduced through the use of ambient concentrations for personal exposures. Models also were created to examine the impact of the fine particle exposures for hours during which the trips occurred. Finally, we conducted sensitivity analyses for all models to test the impact of influential points on our analyses. All effect estimates (β) and their 95% confidence intervals (CI) were transformed into percent changes and reported per interquartile range (IQR) of a pollutant using the formula (e^(β*IQR)^-1) × 100.

## Results

### Study participants and levels of FE_NO_

A total of 25 trips were conducted over the duration of the study, with most subjects (35 of 44) participating in four trips. From our 158 completed person-trips, 299 of the 316 possible samples had sufficient exhaled breath volume for analysis. Summary statistics for these samples are presented by subject characteristics in Table 1. The median FE_NO_ level was 11.1 ppb with a range of 3.7–43.8 ppb in our predominantly Caucasian female population. Although approximately 10% of the subjects reported a doctor diagnosis of asthma and a different 10% reported a diagnosis of chronic obstructive pulmonary disease, tests of differences between these subgroups did not detect any significant differences in FE_NO_ levels at the 95% confidence level.

### Comparison of FE_NO_ and pollution levels before and after trip

Concentrations of PM_2.5_, black carbon, fine particle counts, and coarse particle counts were systematically higher aboard the bus and during the trips than during periods spent at the living facilities. Black carbon, a common indicator for traffic, was most strongly enhanced by the bus trips, with a 9-fold increase in the mean concentration during bus periods. Concentrations were similarly elevated 2- to 3-fold during activity and lunch periods in the urban environment. PM_2.5_, fine particle count, and coarse particle count concentrations also were elevated aboard the bus and during the remainder of the trips, with 2- to 10-fold increases over concentrations measured at the living facilities. Further detail regarding the influence of the bus trips is presented elsewhere (Adar et al. 2007).

Elevated particle levels during the trips also affected the daily mean concentrations measured in the microenvironment, resulting in statistically significant increases in black carbon and coarse particle count concentrations and nonsignificant increases in PM_2.5_ and fine particle count concentrations (Table 2). Despite the increased particle concentrations, levels of FE_NO_ were statistically lower in samples collected the morning after the trip than in samples collected before the trips. No significant differences were found between average ambient particle or gas concentrations between pre- and post-trip days. Correlations were high between all fine particle measures in the microenvironment, with nearly perfect correlation (*r* = 0.98) between the 24-hr mean PM_2.5_ and fine particle count concentrations on days before and after the trips.

Correlations with microenvironmental black carbon were slightly lower but remained strong, with correlations of 0.74 and 0.81 for PM_2.5_ and fine particle counts, respectively. Coarse particles, on the other hand, were weakly correlated with all measures of microenvironmental fine particle concentrations except for black carbon on the day before the trip (*r* = 0.60). Similar to microenvironmental samples, concentrations of PM_2.5_ and black carbon in ambient air were strongly correlated (*r* = 0.74). Weak correlations were observed between ambient particles and ambient gases, but moderate correlation was seen between ambient and microenvironmental fine particles. Ambient gases generally had low to moderate correlations with microenvironmental particles.

### Associations between pollution and FE_NO_ in pre-trip samples

Significant positive associations were consistently found between pre-trip samples of FE_NO_ and levels of microenvironmental fine particulate matter (Table 3). These associations were strongest for PM_2.5_ and fine particle count concentrations, with an approximate 20% increase in FE_NO_ per 4.2 μg/m^3^ or 45 pt/cm^3^ increase in the preceding 6-hr mean pollution level, respectively. Positive associations also were observed with the microenvironmental black carbon whereas generally null associations were found with microenvironmental coarse particle counts. Ambient PM_2.5_ concentrations also exhibited strong positive associations with FE_NO_ in pre-trip samples with a 15% (95% CI, 3–28%) increase predicted per 12.7-μg/m^3^ increase in the 6-hr average and a 22% (95% CI, 7–39%) increase predicted per 9.8-μg/m^3^ increase in the 24-hr average. No associations were found for ambient black carbon or ambient gases (results not shown) in FE_NO_ samples collected before the trips.

### Associations between pollution and FE_NO_ in post-trip samples

On the mornings after the trips, statistically significant associations were again found between measured concentrations of FE_NO_ and all fine particle concentrations measured in the microenvironment (Table 3). Unlike the day before the trip, however, these associations were found with the 24-hr moving averages but not the 6-hr moving averages. In addition, FE_NO_ concentrations were now most strongly associated with microenvironmental black carbon concentrations with a 25% (95% CI, 8–44%) increase in FE_NO_ predicted per increase of 451 ng/m^3^. Associations with the daily microenvironmental PM_2.5_, black carbon, and fine particle count concentrations remained similar in magnitude to those before the trip, but coarse particle count concentrations became significantly and negatively associated with post-trip FE_NO_. These inverse associations were unaffected by control for PM_2.5_ in a two-pollutant model. Associations between ambient PM_2.5_ and FE_NO_ collected after the trips also differed substantially from pre-trip associations. Despite strong and significant associations with pre-trip FE_NO_, associations with post-trip FE_NO_ were virtually nonexistent. Again, ambient gases did not predict levels of FE_NO_ (results not shown).

Further investigation of post-trip FE_NO_ indicated that exposures measured during the trip periods were strongly and significantly associated with FE_NO_ levels (Table 4). These associations were larger than those reported in Table 3 for the 24-hr average as a result of elevated interquartile ranges during the trip periods although the unadjusted effect estimates were similar in magnitude.

### Sensitivity of results

Associations between particulate exposures and FE_NO_ collected before the trip were not the result of a few influential points. On the other hand, associations between microenvironmental particulate pollution and FE_NO_ in samples collected after the bus trips were found to be sensitive to the highest and lowest pollution days. Removing the lowest and highest two trips by exposure (approximately 15% of the data) resulted in nonsignificant relationships between all particles and FE_NO_ on the day after the trip. No explanatory factors were found for the observations at the highest or lowest levels of pollution despite consideration of many factors such as susceptible individuals, weather, wind direction, activity during the trips, facility, and gaseous co-pollutants.

## Discussion

In this investigation we found that fine particulate matter was positively associated with FE_NO_ in samples collected before and after group trips aboard a diesel bus. Although similar effect estimates for microenvironmental exposures before and after the bus trip did not provide evidence of enhanced toxicity for fresh traffic-related particles, our findings do suggest that exposures to traffic increase the levels of FE_NO_ and possibly pulmonary inflammation. This is indicated by our study design with its enhanced exposures to traffic-related particles as well as the strong post-trip associations with concentrations of the traffic-related pollutant black carbon and exposures during the trips. Further support is provided by the fact that 6-hr moving averages of microenvironmental pollution, which do not include the contribution of the bus, were not associated with post-trip FE_NO_. Similarly, ambient pollution was predominantly predictive of FE_NO_ in samples collected pre-trip, indicating that direct exposures to traffic can substantially modify the predictive power of pollution measured at a central location.

Although the clinical implications of these results are uncertain, the findings of this investigation provide further evidence of the proposed inflammatory mechanism through which air pollution may affect health. Evidence of an inflammatory response in human lungs is important partly because it has been hypothesized that pulmonary inflammation may lead to a systemic cascade that can ultimately affect the cardiovascular system. Despite the complexity of roles played by nitric oxide in the body, we selected FE_NO_ as a marker of pulmonary inflammation because it is known to be expressed by macrophages, neutrophils, endothelial cells, and muscle cells as a result of induction by proinflammatory cytokines (Ricciardolo 2003). Elevated FE_NO_ has also been documented for inflammatory diseases such as asthma, rhinitis, viral respiratory tract infections, systemic lupus erythematosis, and acute lung allograft rejections (American Thoracic Society 2006).

Past experimental investigations have demonstrated such effects following direct exposures to diesel exhaust particles. One study found increased constitutive nitric oxide synthase in the airway epithelium and increased inducible nitric oxide synthase in macrophages of mice exposed to diesel exhaust particles compared with control mice (Lim et al. 1998). In addition, l-arginine, an inhibitor of inducible nitric oxide synthase, was found to reduce airway inflammation following exposure to diesel exhaust particles (Lim et al. 1998; Takano et al. 1999).

Human studies also have demonstrated positive associations between exposures to ambient particulate pollution and FE_NO_ in both adults (Adamkiewicz et al. 2004; Jansen et al. 2005; van Amsterdam et al. 1999) and children (Fischer et al. 2002; Koenig et al. 2003; Steerenberg et al. 2001, 2003), with several publications suggesting an increased importance of mobile source pollution. For example, several studies from the Netherlands have reported that traffic-related pollutants such as black smoke, carbon monoxide, nitric oxide, and nitrogen dioxide have associations of similar and sometimes larger magnitude with FE_NO_ than those of PM_10_ (Fischer et al. 2002; Steerenberg et al. 2001; van Amsterdam et al. 1999). The magnitudes of these associations with traffic-related pollutants seem to be generally consistent across this and other studies, suggesting that these associations may be similar in older populations and children. One study did imply, however, that proximity to traffic might be an effect modifier of the associations with FE_NO_: Children living in urban areas had a more profound response to these pollutants than those in suburban areas (Steerenberg et al. 2001). We did not find, however, that bus trips resulted in obvious increases in toxicity for PM_2.5_ and fine particle counts.

In this investigation, positive associations with FE_NO_ were consistently found for fine particle concentrations. Negative associations were generally found with coarse particle concentrations and no associations were found for ambient gas concentrations. Although no other observational studies examined associations with coarse particles, our findings are supported by one experimental study in which humans exposed to concentrated coarse particles did not elicit a response with respect to FE_NO_ (Gong et al. 2004). On the other hand, several investigations have found associations between FE_NO_ and ambient gases (Fischer et al. 2002; Nickmilder et al. 2003; Steerenberg et al. 2001, 2003; van Amsterdam et al. 1999). Our lack of associations may be attributable to the poor correlation in this data set between ambient gases and microenvironmental particles. Although most other investigators did not report the correlations between particles and gases, it is possible that correlations were higher for those studies such that confounding might exist. In the only investigation that used multipollutant models, ambient nitric oxide was found to lose its significance following control for PM_2.5_, although the effect estimate for PM_2.5_ remained strong and significant (Adamkiewicz et al. 2004). Similarly, no associations were found between controlled ozone exposures in humans and FE_NO_ in an experimental study design (Olin et al. 2001). Nevertheless, gases may have a causal connection to pulmonary inflammation; respiratory outcomes have been documented in humans following controlled exposures to nitrogen dioxide and sulfur dioxide (Tunnicliffe et al. 2003).

In several previous investigations, associations between air pollution and FE_NO_ appeared to be limited to specific susceptible subgroups, such as asthmatics. Therefore, it is also possible that characteristics of our study population may explain differences between this and previous studies with respect to gaseous exposures. For example, associations between air pollution and FE_NO_ in Steubenville, Ohio, were limited predominantly to senior adults with chronic obstructive pulmonary disease (Adamkiewicz et al. 2004). In Seattle, Washington, associations were limited to asthmatic children who were not using inhaled corticosteroids (Koenig et al. 2003). In our investigation, associations between fine particles and inflammation were relatively homogeneous across participants without evidence of effect modification by respiratory disease or medication use. Similarly, we found no enhanced susceptibility for individuals with diabetes, obesity, or elevated circulating inflammatory markers despite the fact that such susceptibility was previously found in this population for systemic inflammation (Dubowsky et al. 2006). Despite these apparent differences, our and other investigations all had few potentially susceptible individuals (< 10), making it difficult to draw conclusions regarding true effect modification.

The relatively low numbers of study subjects (44) and total number of trips conducted (25) can be considered a general limitation of this study. For example, although strong and statistically significant associations between fine particulate pollution and FE_NO_ were found post-trip, two influential points were found to drive these associations. Although investigation of these 2 sampling days did not reveal any obvious reason for exclusion, it is possible that some unidentified aspect of the bus trip, such as social activity or exercise, positively affected our subjects, because the crude levels of FE_NO_ generally decreased after the trips. The generalizability of these results also may be somewhat limited by the predominance of Caucasian women, though the proportion of elderly women does exceed the proportion of elderly men in the general U.S. population. Despite these potential limitations, robust significant associations found pre-trip, consistency between measures of microenvironmental pollution on the 2 days, and similarity to past studies support the overall findings of this investigation.

In summary, this study links increasing levels of fine particulate matter to increasing FE_NO_ in older adults, which is suggestive of elevated airway inflammation. Although associations with both microenvironmental and ambient exposures were evident in samples collected after routine exposures at home, samples collected after periods spent aboard a diesel bus exhibited associations only with measures of microenvironmental exposure. Strong associations were also observed for exposures during trip periods. Associations following the trip may have been sensitive to 2 influential days, but associations before the trip seemed to be reasonably robust.

## Figures and Tables

**Table 1:** FE_NO_ levels by participant characteristics.

Characteristic	Subjects [*n* (%)]	Samples [*n* (%)]	Median (ppb)	Range (ppb)
All participants	44 (100)	299 (100)	11.1	3.7–43.8
Sex				
Female	36 (82)	245 (82)	11.6	3.7–43.8
Male	8 (18)	54 (18)	9.9	3.7–24.9
Race/ethnicity				
White	41 (93)	280 (94)	11.5	3.7–43.8
African American	3 (7)	19 (6)	8	4.4–37.4
Age (years)				
62–79	20 (45)	134 (45)	11.4	3.7–43.8
80–94	24 (55)	165 (55)	11	3.7–37.4
Cigarette smoking^a^				
Never	23 (52)	157 (53)	10.8	3.7–43.8
Former	21 (48)	142 (47)	11.6	3.7–37.1
Asthma^b^				
Yes	4 (9)	25 (8)	10.8	6.2–37.4
No	40 (91)	274 (92)	11.2	3.7–43.8
COPD^b^				
Yes	5 (11)	38 (13)	10.4	3.7–43.8
No	39 (89)	261 (87)	11.2	3.7–37.4

COPD, chronic obstructive pulmonary disease.

a All subjects were current nonsmokers.

b As defined by report of doctor diagnosis by participant.

**Table 2:** FE_NO_ and 24-hr mean environmental levels preceding health measurements—before (pre) and after (post) trips.

	Pre-trip		Post-trip		
	Median	IQR	Median	IQR	Pre vs. post *p*-value^a^
FE_NO_ (ppb)	12.1	9.0–15.8	10.2	7.8–13.9	0.0002
Microenvironmental measures					
PM_2.5_ (μg/m^3^)	7.6	6.4–9.8	9.2	7.2–11.3	0.14
Black carbon (ng/m^3^)	283	194–361	681	484–969	< 0.0001
Fine particle counts (pt/cm^3^)	41	32–65	56	32–71	0.36
Coarse particle counts (pt/cm^3^)	0.02	0.01–0.04	0.09	0.07–0.1	< 0.0001
Room air nitric oxide (ppb)	12.4	6.7–21.9	11.3	6.4–30.4	0.71
Apparent temperature (°C)	21.5	20.5–22.0	20.7	19.8–22.7	0.89
Ambient measures					
PM_2.5_ (μg/m^3^)	14.8	11.2–20.1	16.5	11.7–21.6	0.94
Black carbon (ng/m^3^)	795	678–988	925	729–1,127	0.59
Carbon monoxide (ppm)	0.4	0.3–0.5	0.4	0.4–0.5	0.85
Nitrogen dioxide (ppb)	15.5	12.9–19.4	18.3	14.7–21.2	0.30
Sulfur dioxide (ppb)	3.6	2.8–6.1	2.9	2.1 –6.0	0.52
Ozone (ppb)	24.3	17.5–31.5	25.3	16.9–29.7	0.84
Pollen counts (pt/m^3^)	92.5	42–390	102	48–346	0.82
Mold counts (pt/m^3^)	7,599	2,720–23,070	11,047	3,600–19,970	0.57

a Differences between pre-trip and post-trip FE_NO_ values were calculated using a crude mixed-model with random subject effects. Differences between exposure and meteorologic parameters were determined using a crude regression model that controlled for autoregressive terms.

**Table 3:** Percent change (95% CI) in FE_NO_ per interquartile change in microenvironmental and ambient particles.

	Averaging period (hr)	Mean IQR	Pre-trip percent change	Post-trip percent change
Microenvironmental particles				
PM_2.5_ (μg/m^3^)	6	4.2	17.0 (6.7 to 28.2)**	−1.7 (−13.8 to 12.2)
	24	4.1	12.5 (2.1 to 24.0)*	13.0 (5.4 to 21.0)**
Black carbon (ng/m^3^)	6	251	6.9 (0.3 to 13.9)*	−7.6 (−18.0 to 4.2)
	24	451	24.9 (−0.05 to 56.1)	24.8 (8.0 to 44.4)**
Fine particle counts (pt/cm^3^)	6	45	20.2 (6.2 to 36.0)**	3.5 (−7.3 to 15.6)
	24	39	15.5 (3.7 to 28.7)*	12.9 (5.3 to 21.0)**
Coarse particle counts (pt/cm^3^)	6	0.03	−4.5 (−14.4 to 6.4)	−3.9 (−12.0 to 5.0)
	24	0.07	0.7 (−21.8 to 29.8)	−25.8 (−33.7 to −16.8)**
Ambient particles				
PM_2.5_ (μg/m^3^)	6	12.7	14.9 (3.1 to 28.0)*	−0.5 (−10.3 to 10.4)
	24	9.8	21.9 (6.7 to 39.4)**	−4.7 (−17.1 to 9.6)
Black carbon (ng/m^3^)	6	529	4.0 (−4.5 to 13.3)	13.9 (2.5 to 26.5)*
	24	350	−2.1 (−13.6 to 10.8)	6.3 (−5.3 to 19.3)

* Statistical significance at the 95% confidence level.

** Statistical significance at the 99% confidence level. All models were controlled for random subject effects, trip type, day of week, collection device, vitamin use, pollen, mold, apparent temperature in the current hour, time between collection and analysis, and room air nitric oxide.

**Table 4:** Percent change (95% CI) in post-trip levels of FE_NO_ per interquartile change in microenvironmental concentrations measured during the trip.

	IQR	Post-trip percent change
PM_2.5_ (μg/m^3^)	8.7	24.4 (15.1 to 34.4)*
Black carbon (ng/m^3^)	1,245	26.5 (7.0 to 49.6)*
Fine particle counts (pt/cm^3^)	60	18.3 (10.4 to 26.9)*
Coarse particle counts (pt/cm^3^)	0.15	−16.0 (−21.5 to −10.1)*

All models were controlled for random subject effects, trip type, day of week, collection device, vitamin use, pollen, mold, apparent temperature in the current hour, time between collection and analysis, and room air nitric oxide.

* Statistical significance at the 99% confidence level.
